# Temporomandibular Disorders and Vitamin D Deficiency: What Is the Linkage between These Conditions? A Systematic Review

**DOI:** 10.3390/jcm11216231

**Published:** 2022-10-22

**Authors:** Martina Ferrillo, Lorenzo Lippi, Amerigo Giudice, Dario Calafiore, Teresa Paolucci, Filippo Renò, Mario Migliario, Leonzio Fortunato, Marco Invernizzi, Alessandro de Sire

**Affiliations:** 1 Dentistry Unit, Department of Health Sciences, University of Catanzaro “Magna Graecia”, 88100 Catanzaro, Italy; 2 Physical and Rehabilitative Medicine, Department of Health Sciences, University of Eastern Piedmont “A. Avogadro”, 28100 Novara, Italy; 3 Translational Medicine, Dipartimento Attività Integrate Ricerca e Innovazione (DAIRI), Azienda Ospedaliera SS. Antonio e Biagio e Cesare Arrigo, 15121 Alessandria, Italy; 4 Physical Medicine and Rehabilitation Unit, Department of Neurosciences, ASST Carlo Poma, 46100 Mantova, Italy; 5 Physical Medicine and Rehabilitation, Department of Oral, Medical and Biotechnological Sciences, Physical Medicine and Rehabilitation, University G. D’Annunzio, 66100 Chieti, Italy; 6 Innovative Research Laboratory for Wound Healing, Health Sciences Department, Università del Piemonte Orientale, 28100 Novara, Italy; 7 Dentistry Unit, Department of Translational Medicine, University of Eastern Piedmont, 28100 Novara, Italy; 8 Physical Medicine and Rehabilitation Unit, Department of Medical and Surgical Sciences, University of Catanzaro “Magna Graecia”, 88100 Catanzaro, Italy

**Keywords:** temporomandibular disorders, rehabilitation, oral health, oral hygiene, vitamin D, vitamin D deficiency

## Abstract

Although a growing body of literature has been emphasizing the role of vitamin D in oral health, there is still a gap of knowledge regarding the correlation between temporomandibular disorders (TMDs) and vitamin D. Therefore, the aim of this systematic review was to assess the linkage between hypovitaminosis D and TMDs to map the current literature in this field. On 10 September 2022, PubMed, Scopus, and Web of Science databases were systematically searched from the date of their inception to identify the studies that had assessed patients with TMDs. The primary outcome assessed in this review was the relationship between hypovitaminosis D and TMDs. Out of the 329 studies identified, 13 studies met the eligibility criteria and were included in the present work. Seven studies assessed the relationship between vitamin D and TMDs, reporting that vitamin D serum levels are lower in patients with TMDs. Our results suggested that vitamin D receptor (VDR) polymorphisms might have a role in TMDs’ development. However, the quality assessed underlined that only one study did not present a serious risk of bias. Further good-quality studies are needed to clarify the linkage between vitamin D deficiency and TMDs, but the evidence currently available has suggested potential correlations.

## 1. Introduction

Temporomandibular disorders (TMDs) are a set of clinical problems involving the masticatory musculature, the temporomandibular joint (TMJ), surrounding structures, or combinations of these components [1]. Typical signs and symptoms of TMDs are facial pain, clicking or crepitus of the TMJs, limited jaw movement capacity, and a deviation in the movement patterns of the mandible [2]. Subsequently, chronic pain, joint noise, restriction of mandibular range of motion (ROM), and functional difficulty may also develop [3]. According to the diagnostic criteria for TMDs (DC/TMD), Axis I, TMDs can be divided into muscle disorders (Group I); intra-capsular disorders, including disc displacement (Group II); and arthralgia, arthritis, and arthrosis (Group III) [1,4]. Although TMDs are considered to be a sub-classification of musculoskeletal disorders, the aetiological factors are not clearly understood, but are thought to involve anatomical factors. Mechanical displacement; prolonged use of mastication muscles; altered skeletal maturity; malocclusion; repetitive trauma at the TMJ; psychological disorders (e.g., anxiety and depressive syndrome); postural deviation; trauma; and general hypermobility of the joints could be potential risk factors [5,6,7,8,9,10]. However, it should be taken into consideration that a painful TMD might be considered as one of the main causes of orofacial pain [11,12], in the case of the exclusion of odontogenic causes [13,14,15].

Vitamin D is an important component in calcium homeostasis, which is known to have a key role in bone health, including articular structures and muscles [16,17]. Vitamin D deficiency is prevalent worldwide and approximately one billion people may have low vitamin D levels [18]. This condition could be associated with a number of disorders involving bone, cartilage, and the associated tissues. Furthermore, it could lead to reduced muscle strength and poor physical performance, and studies have shown an association between low vitamin D status and musculoskeletal disorders, such as osteoarthritis, risk of falling, and osteoporosis [16,17,19,20,21,22]. Moreover, among the several genes that have been associated with TMDs, the vitamin D receptor (VDR) gene is regarded as one of the most important candidate genes for investigating the genetic factors that contribute to the pathophysiology of TMDs [23]. Furthermore, on the one hand, studies [24,25] have related the receptor activator of the NF-κB ligand (RANKL)/RANK/osteoprotegerin (OPG) system to vitamin D; however, on the other hand, an increased RANKL/OPG ratio in the subchondral bone has appeared during the early stages of TMJ osteoarthritis [26,27].

Lastly, vitamin D might have a role in regulating MAP kinases and inhibiting the NF-kB signaling pathway, with intriguing implications for oxidative stress and inflammation [28,29,30]. To date, although vitamin D has been suggested as an effective non-pharmacological intervention to counteract systemic inflammation and manage bone health [31,32,33], there is still a large gap of knowledge about the role of vitamin D in musculoskeletal disorders.

Therefore, the aim of this systematic review was to assess the linkage between hypovitaminosis D and TMDs to provide a broad overview of the current evidence in this field and to guide future research in the identification of the potential therapeutic effects of vitamin D supplementations.

## 2. Materials and Methods

### 2.1. Registration and Search Strategy

This systematic review was performed following the preferred reporting items for the systematic reviews and meta-analyses (PRISMA) statement [20]. We defined the study protocol before study initiation and it was submitted to the international prospective register of systematic reviews on 15 May 2022, and accepted on 23 May 2022 (PROSPERO, https://www.crd.york.ac.uk/prospero accessed on 14 October 2022; registration number: CRD42022332980).

PubMed, Scopus, Web of Science, and Cochrane Central Register of Controlled Trials (CENTRAL) were systematically searched for relevant studies, published from inception until 25 August 2022. Table 1 shows the search strategy adopted for each database in detail.

The reference lists of all the relevant studies were screened for other studies that were potentially suitable for the present review. The study assessment was performed independently and synchronously by two different authors.

### 2.2. Selection Criteria

After duplication removal, two independent reviewers screened all potential records for title and abstract. Any disagreement was resolved through discussion between the two reviewers or by consultation with a third reviewer. The selected articles were subsequently screened in full text by the two authors. In case of disagreement, a consensus was achieved by discussion, or by the decision of a further experienced reviewer.

The following PICO model was used to assess the eligibility the relevant studies:

(P) Participants: patients with a TMD were assessed.

(I) Intervention: no restrictions in terms of therapeutic intervention were adopted.

(C) Comparator: any comparator, including healthy subjects, e.g., patients with normal values of vitamin D, or patients without diagnosis of a TMD.

(O) Outcome: the primary outcome was the relationship between hypovitaminosis D and TMDs. The secondary outcomes were as follows: (i) the relationship between vitamin D serum levels, physical and psychological impairment, and quality of life in patients with dental diseases; (ii) the relationship between dental diseases, vitamin D serum levels, and biomarkers expression; and (iii) genotype and frequency of vitamin D receptor (BsmI, Apa1, and Taq1).

We included any relevant studies without restriction in study design. We excluded the following: (1) studies written in a language different from English; (2) participants with pathological disorders affecting calcium homeostasis (i.e., hypoparathyroidism and sarcoidosis); (3) studies on animals; and (4) full-text unavailability (i.e., posters and conference abstracts).

### 2.3. Data Extraction and Synthesis

All the studies included were assessed by two different authors and the data were extracted independently. Any disagreement between the two reviewers was solved by collegial discussion. A third reviewer was asked in case of further disagreement.

The following data were extracted: (1) title, (2) authors, (3) publication year, (4) nationality, (5) participants’ characteristics, (6) interventions’ characteristics, (7) comparator characteristics; and (8) study results.

A qualitative method was used to synthesize the data extracted. Text and tables have been used to provide a descriptive summary and explanation of study characteristics and main findings.

Subgroup analyses were performed based on the patients’ characteristics and the outcomes assessed.

### 2.4. Quality Assessment and Risk of Bias

The quality of the studies included were assessed using the Joanna Briggs Institute Critical Appraisal Checklist [34]. Two authors assessed the study quality of the included papers. Discordance between the reviewers was solved by collegial discussion. In case of disagreement, a third reviewer was asked.

## 3. Results

### 3.1. Study Characteristics

**Figure 1:**
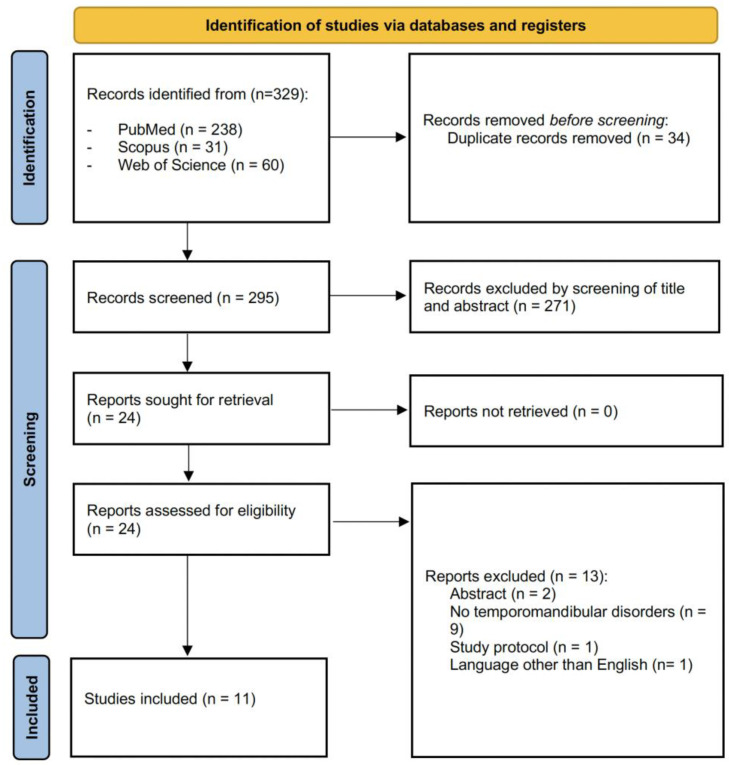
PRISMA 2020 flow diagram.

The search strategy was performed on 10 September 2022 and it identified 329 records from the three databases. Figure 1 shows the PRISMA flow diagram of the search process. After duplication removal, 295 studies were assessed for eligibility and screened for the title and abstract. After the exclusion of 271 records, 24 full-text records were assessed for eligibility. Thirteen articles were excluded for inconsistency with the eligibility criteria (two records were abstract, nine records did not evaluate TMD, one record was a study protocol, and one record was excluded for being in a language other than English). As a result, 11 studies were included in the present work.

The publication year of the studies that were included in this review ranged between 2017 [35,36] and 2022 [37]. The nationalities of the studies included in this review were as follows: seven studies (69%) were conducted in Asia (two in Iran [38,39], three in India [36,37,40], one in Iraq [35], and one in Korea [41]); one study (8%) was conducted in Europe (in Norway [42]); and the remaining three studies (23%) were Turkish [43,44,45].

Seven were cross-sectional studies [35,36,37,38,39,42,45], two had a prospective observational design [41,43], one was a prospective case-control study [44], and one study was a randomized controlled trial [40].

### 3.2. Participants’ Characteristics

In the present review, 1267 human subjects were assessed. Out of them, 607 were included in the intervention groups and 560 were included in the control groups. Most of the human subjects were female (*n*: 662), while 244 were male. The studies conducted by Bashir et al. [37], Gupta et al. [40], and Yilmaz et al. [45] did not characterize the sample for gender. The mean ages of the subjects who were included in this study ranged from 8.50 ± 43.02 years [35] to 57.2 ± 4.6 years [41]. Further details are shown in Table 2.

### 3.3. Vitamin D Serum Levels

Seven papers [35,38,39,41,42,43,44] assessed the relationship between hypovitaminosis D and TMDs. In the study by Ahmed et al. [35], TMD patients with rheumatoid arthritis were assessed. The results showed that the vitamin D serum levels were significantly lower in TMD patients (*p* = 0.001). Hong et al. [41] assessed young and post-menopausal females with temporomandibular joint (TMJ) osteoarthritis (OA), reporting a significant association between the progression of the TMJ OA and the vitamin D levels (*p* = 0.045) of the subjects. Nemati et al. [39] showed a significant difference in the serum levels of vitamin D between patients with TMDs and the control groups (*p* = 0.001), reporting a higher prevalence of vitamin D deficiency in patients with a TMD. Yildiz et al. [44] showed that the serum vitamin D level were significantly different between the patient and the control group (*p* = 0.008). In particular, a serious vitamin D deficiency was more prevalent in patients with a TMD (*p* = 0.00001). Moreover, Demir et al. [43] and Madani et al. [38] did not report significant differences in the vitamin D serum levels between the TMD patients and the healthy subjects. In contrast, Staniszewski et al. [42] showed that TMD patients had significantly higher serum levels of vitamin D (*p* = 0.005) compared to the controls.

### 3.4. Genotype and Frequency of Vitamin D Receptor Polymorphisms

Three studies [37,44,45] assessed the genotype and the frequency of VDR polymorphisms (BsmI, Apa1, and Taq1). Bashir et al. [37] showed that the BsmI polymorphism was significantly higher for the mutant genotype/allele in the TMD patients (*p* = 0.0015), while a significant association between the TMJ internal derangement development and the allelic and genotypic frequencies of BsmI. Moreover, in the study by Yildiz et al. [44], logistic regression showed that the bb genotype and b allele carriers of the VDR BsmI polymorphism were significantly associated with an increased risk of disc dislocation (*p* = 0.022 and *p* = 0.01, respectively). The VDR BsmI BB genotype was higher in the control group compared to the TMD patients (*p* = 0.045). Yilmaz et al. [45] reported that TMJ internal derangement was associated with the Taq1 polymorphism (OR: 0.63) and the Tt genotype (OR: 0.44-fold), without reaching the significance value (*p* > 0.05). In patients with TMJ internal derangement, the Tt and tt genotypes had odds ratios of 0.53 and 0.73, respectively (*p* > 0.05). Combined VDR genotypes revealed that AATT had a 3.3-fold (*p* = 1.21) odds ratio, while AATt had a 2.0-fold odds ratio (*p* = 0.29) (OR: 0.59, 95% CI 0.23–1.49, *p* = 0.26) compared to AaTt. When the TMD patients were compared with the healthy subjects, the Apa1 Aa genotype, compared to the AA genotype, had odds ratios of 1.65, 1.79 and 1.64, respectively (*p* > 0.05).

### 3.5. Vitamin D Serum Levels and Biomarkers Expression

Three studies [35,42,43] assessed the relationship between vitamin D serum levels, biomarkers expression, and TMDs.

A more detailed study by Ahmed et al. [35] showed a significant increase in the total ALP and IL-1 serum levels in TMD patients (*p* = 0.01), with a significant negative correlation between serum vitamin D activity and ALP (*p* = 0.001), and between serum vitamin D activity and IL-1 (*p* = 0.001). Moreover, Demir et al. [43] showed that only parathyroid hormone serum levels were significantly higher in patients with a TMD, compared with the control group (*p* < 0.001). In addition, Staniszewski et al. [42] showed that TMD patients had significantly higher values of hemoglobin (*p* = 0.036), cobalamin (*p* = 0.023), albumin (*p* = 0.005), and parathyroid hormone (*p* = 0.038), with lower values of creatinine (*p* = 0.006) and potassium (*p* = 0.011).

### 3.6. Vitamin D Serum Level, Physical Impairment, Psychological Impairment, and Quality of Life

In two studies [36,40], the relationship between vitamin D serum levels and physical impairment, psychological impairment, and quality of life were assessed. In particular, Gupta et al. [40] showed a significant difference in comfort mouth opening, the visual analogue scale score, and maximum mouth opening (*p* < 0.05) in TMD patients who were treated with a stabilization splint and vitamin D supplementation. Moreover, in the study by Khanna et al. [36], low serum vitamin D levels were associated with TMJ pain and/or discomfort, which had a significant negative impact on the various ADLs of the participants.

### 3.7. Quality Assessment and Risk of Bias

Two authors assessed the quality of the studies included in this review independently. Any discordance between the reviewers was solved by collegial discussion. In the case of a disagreement, a third reviewer was asked.

The quality assessment was performed following the Joanna Briggs Institute Critical Appraisal Checklist for Randomized Controlled Trials and the JBI Critical Appraisal Checklist for Quasi-Experimental Studies. One study [40] did not present a serious risk of bias. In contrast, the remaining studies that were included in this review [35,36,37,38,39,41,42,43,44,45] presented at least one serious risk of bias, which translated into an overall serious risk of bias for that study. The main quality concerns included the lack of data on the baseline characteristics of the studies’ participants, nonrandom sampling approaches (convenience samples), missing data, and the lack of a reliable tool to estimate and report outcomes.

Further details about the quality assessment of each study included in this systematic review are shown in Table 3.

## 4. Discussion

The aim of this systematic review was to assess the correlation between hypovitaminosis D and TMDs, to provide a broad overview of the current evidence in this field and to guide future research in the identification of the potential therapeutic effects of vitamin D supplementations.

As the etiopathogenesis of TMDs is not completely understood, the therapeutic options are not always successful [46,47,48,49,50]. Therefore, understanding the etiology, and identifying and eliminating the potential pathogenic factors could make a significant contribution to the knowledge about TMDs.

The studies included in this systematic review [35,38,39,41,42,43,44] showed that the vitamin D serum levels were significantly lower in TMD patients, compared to the healthy subjects. In 2022, Gupta et al. [40] conducted an RCT and evaluated the efficacy of vitamin D supplementation plus stabilization splint therapy, in TMD patients. The authors showed that the experimental group reported a significant improvement in the VAS score, comfortable mouth opening, and maximum mouth opening.

It has been shown that vitamin D plays a significant role in musculoskeletal disorders and that vitamin D deficiency can cause bone loss, hypocalcemia, and poor muscle strength, manifested by musculoskeletal pain [51]. Moreover, vitamin D seems to have a role in pain intensity and in the management of pain in varying clinical settings [52]. In this context, Wu et al. [53] conducted a systematic review to determine if vitamin D supplementation could reduce pain scores when compared with the placebo. The authors included 19 RCTs and concluded that vitamin D supplementation could have a role in the management of chronic pain. This concept must be taken into account since muscular TMDs could be attributed to common dysfunctions of the central pain regulation mechanisms (central sensitization), and could be associated with the development of craniofacial allodynia [54]. In this context, Khanna et al. [36] compared TMD patients with vitamin deficiency to TMD patients with average vitamin D serum levels. The authors showed that low serum vitamin D levels were associated with TMJ pain and/or discomfort, with a negative impact on the various activities of the daily living of the participants.

The biological impact of the active form of vitamin D occurs after its binding to VDR, which is a member of the steroid hormone receptor family [55]. More than 100 restriction polymorphic sites have been shown in the VDR gene and ApaI (rs7975232)—TaqI (rs731236), BsmI (rs1544410), and FokI (rs2228570) are the best known ones [56]. Three studies [37,44,45] assessed the genotype and frequency of vitamin D receptor polymorphisms in patients affected by arthrogenous TMD. A more detailed study by Bashir et al. [37] showed that the BsmI polymorphism was significantly higher for mutant genotype/allele in TMD patients, and Yildiz et al. [44] showed that the bb genotype and b allele carriers of the VDR BsmI polymorphism were significantly associated with an increased risk of disc dislocation. On the other hand, Yilmaz et al. [45] reported that TMJ internal derangement was associated with the Taq1 polymorphism and Tt genotype, without reaching the significance value. It seems that arthrogenous TMD may be a rather localized phenomenon.

In this scenario, previous studies have underlined the potential role of VDR polymorphisms in musculoskeletal disorders. In a more detailed study, the recent case-control study by Colombini et al. [57] underlined that three genetic VDR variants—BsmI, ApaI, and TaqI—might be associated with lumbar spine pathologies, emphasizing the role of genotypes/alleles/haplotypes screening in the clinical assessment of musculoskeletal disorders. Accordingly, the recent systematic review by Azharuddin et al. [58] identified some individual studies that supported that VDR polymorphisms rs731236 might be associated with herniated nucleus pulposus, suggesting the potential development of a precise assessment of individual phenotypes in the integrated therapeutic management of musculoskeletal disorders. Despite this evidence, none of the studies included in our systematic review assessed the mechanisms that underpin the relationship between TMDs and VDR. However, specific VDR expressions might have a role in vitamin D supplementation therapies, as supported by previous studies [59,60].

On the other hand, to the best of our knowledge, this is the first systematic review that has assessed the role of VDR polymorphisms in patients with TMDs. Our results might pave the way for future research studies, which focus on specific VDR polymorphisms, and which address the role of vitamin D supplementation in more responsive patients to optimize the therapeutic management of TMD patients and to focus resources on the most effective therapies, specific to phenotypes.

Despite these considerations, we are aware that this study was not free from limitations. In this context, the main limitation of this review was about the quality of the studies included. Just one study was a randomized controlled trial and the other studies that were included raised some concerns in the quality assessment. Moreover, the lack of a quantitative synthesis represents one other concern about this work.

On the other hand, it should be noted that our results underlined the potential relationship between vitamin D serum levels and TMD disorders, emphasizing the needing for future good quality studies to clarify the role of vitamin D in these detrimental conditions.

## 5. Conclusions

Taken together, the results of this systematic review showed that vitamin D serum levels could often be lower in patients with a TMD. Moreover, we highlighted the evidence regarding VDR polymorphisms in patients with a TMD.

In light of these considerations, our data suggested that vitamin D serum levels and VDRs might have a role in TMDs’ onset and progression, despite the fact that the mechanisms underpinning these relationships are far from being fully characterized. Therefore, further good quality studies are needed to clarify the effects of vitamin D supplementation in the comprehensive rehabilitation management of TMDs.

## Figures and Tables

**Table 1:** Search strategy.

**PubMed:** ((("vitamin d"[MeSH Terms] OR "vitamin d"[All Fields] OR "ergocalciferols"[MeSH Terms] OR "ergocalciferols"[All Fields] OR "calcifediol"[MeSH Terms] OR "calcifediol"[All Fields] OR "25 oh d3"[All Fields])) AND ((("temporomandibular joint disorders"[MeSH Terms]) OR ("temporomandibular"[All Fields] AND "joint"[All Fields] AND "disorders"[All Fields]) OR ("osteoarthritis"[MeSH Terms] OR "osteoarthritis"[All Fields] OR "osteoarthritides"[All Fields]) OR ("masticatory muscles"[MeSH Terms] OR "masticatory"[All Fields] AND "muscles"[All Fields]) OR ("masticatory muscles"[All Fields])) AND (("disease"[MeSH Terms] OR "disease"[All Fields] OR "disorder"[All Fields] OR "disorders"[All Fields] OR "disorder s"[All Fields] OR "disordes"[All Fields]) OR ("bruxism"[MeSH Terms] OR "bruxism"[All Fields]))))
**Scopus:** ((("vitamin d" OR "ergocalciferols" OR "calcifediol" OR "25 oh d3")) AND (("temporomandibular joint" OR "temporomandibular joint disorders" OR "TMD" OR "temporomandibular osteoarthritis") OR ("masticatory muscles" OR "masticatory muscles disease" OR "masticatory muscles disorder") OR ("bruxism")))
**Web of Science:** ((("vitamin d" OR "ergocalciferols" OR "calcifediol" OR "25 oh d3")) AND (("temporomandibular joint" OR "temporomandibular joint disorders" OR "TMD" OR "temporomandibular osteoarthritis") OR ("masticatory muscles" OR "masticatory muscles disease" OR "masticatory muscles disorder") OR ("bruxism")))

**Table 2:** Main characteristics of the studies included in the present systematic review.

Authors	Journal	Design	Nationality	Population	Age (years)	Intervention	Comparator	Outcome	Time Points	Main Findings
Ahmed, H.S. et al., 2017 [35]	Biomedical & Pharmacology Journal	Cross-sectional study	Iraq	Total: *n* = 90; M/F = 37/53 Group 1: *n* = 45; M/F = 15/30 Group 2: *n* = 45; M/F = 22/23	Total: N/A Group 1: 12.60 ± 50.58 Group 2: 8.50 ± 43.02	Group 1: TMD patients with rheumatoid arthritis	Group 2: healthy subjects	Calcium, total alkaline phosphatase (ALP) activity, and IL-1 serum levels		The vitamin D serum levels were significantly lower in TMD patients (*p* = 0.001). Moreover, results showed a significant increase in total ALP and IL-1 serum levels in TMD patients (*p* = 0.01). There was a significant negative correlation between serum vitamin D activity with total ALP activity and IL-1 in temporomandibular disorder patients (*p* = 0.001)
Bashir, S. et al., 2022 [37]	Gene Reports	Cross-sectional study	India	Total: *n* = 106; M/F = N/A Group 1: *n* = 53; M/F = N/A Group 2: *n* = 53; M/F = N/A	Total: (18–45) Group 1: N/A Group 2: N/A	Group 1: TMD patients with internal derangement (TMD-ID)	Group 2: healthy subjects	Genotype and frequency of VDR gene polymorphisms for *BsmI.*		*BsmI* polymorphism was significantly higher for mutant genotype/allele in TMD-ID patients than for controls (OR = 4.1; *p* = 0.0015). Therefore, there was a significant association between TMJ internal derangement development and allelic and genotypic frequencies of *BsmI.*
Demir, C.Y. et al., 2018 [43]	Journal of International Medical Research	Prospective observational study	Turkey	Total: *n* = 100; M/F = 23/77 Group 1: *n* = 50; M/F = 11/39 Group 2: *n* = 50; M/F = 12/38	Total: 27.07 ± 5.44 Group 1: 28.24 ± 10.41 Group 2: 30.90 ± 8.49	Group 1: TMD patients	Group 2: healthy subjects	Vitamin D, parathyroid hormone, calcitonin, calcium, phosphorus, and magnesium serum levels.		Only parathyroid hormone serum levels were found to be significantly higher in Group 1 versus Group 2 (*p* < 0.001).
Gupta, A.K. et al., 2022 [40]	The Journal of Indian Prosthodontic Society	Randomized controlled trial	India	Total: *n* = 36; M/F = N/A Group 1: *n* = 18; M/F = N/A Group 2: *n* = 18; M/F = N/A	Total: (18–45) Group 1: N/A Group 2: N/A	Group 1: stabilization splint *plus* vitamin D supplementation in TMD patients with vitamin D serum levels < 30 ng/mL	Group 2: stabilization splint *plus* placebo drug in TMD patients with vitamin D serum levels < 30 ng/mL	Comfort mouth opening, VAS, maximum mouth opening, vitamin D serum levels, TMJ tenderness, efficacy of stabilization splint therapy plus vitamin D supplementation.	T0 (baseline), T1 (1 week), T2 (1 month), T3 (2 months), and T4 (3 months) after therapy	By intergroup analysis, a significant difference was only shown in comfort mouth opening, VAS, and maximum mouth opening (*p* < 0.05).
Hong, S.W. et al., 2021 [41]	Clinical Oral Investigations	Prospective observational study	Korea	Total: *n* = 100; M/F = 0/100 Group 1: *n* = 59; M/F = 0/59 Group 2: *n* = 41; M/F = 0/41	Total: N/A Group 1: 23.4 ± 3.4 Group 2: 57.2 ± 4.6	Group 1: 43 young females with TMD osteoarthritis Group 2: 29 post-menopausal females with TMD osteoarthritis	Group 1: 16 young, healthy subjects Group 2: 12 post-menopausal, healthy subjects	TMD osteoarthritis progression, Vitamin D serum level, and BMD	T0 (baseline) and T1 (12 months)	The baseline levels of 25-dihydroxyvitamin D were significantly different among the control group in the young females. There was a significant association between progression of the TMJ osteoarthritis and Vitamin D levels in the young and post-menopausal females (*p* = 0.045).
Khanna, S.S. et al., 2017 [36]	Journal of Clinical and Diagnostic Research	Cross-sectional study	India	Total: *n* = 100; M/F = 39/61 Group 1: *n* = 50; M/F = 20/30 Group 2: *n* = 50; M/F = 19/31	Total: 48.98 (25–70) Group 1: 52.96 Group 2: 45.0	Group 1: TMD patients with vitamin D serum levels < 30 ng/mL	Group 2: TMD patients with normal vitamin D serum levels	TMD pain and discomfort in the activities of daily living.		Authors showed that low serum vitamin D levels were associated with TMJ pain and/or discomfort, which had a significant (*p* = NR) negative impact on the various activities of daily living of the participants.
Madani, A. et al., 2019 [38]	The Journal of Craniomandibular & Sleep Practice	Cross-sectional study	Iran	Total: *n* = 80; M/F = 14/66 Group 1: *n* = 51; M/F = 5/46 Group 2: *n* = 29; M/F = 9/20	Total: N/A Group 1: 27 (21–46) Group 2: 32 (20–48)	Group 1: TMD patients	Group 2: healthy subjects	Serum concentrations of calcium, phosphate, alkaline phosphatase, parathyroid hormone, and vitamin D.		No statistically significant differences were observed between different groups in any of the variables studied (*p* > 0.05).
Nemati, M. et al., 2021 [39]	Journal of Maxillofacial and Oral Surgery	Cross-sectional study	Iran	Total: *n* = 110; M/F = 39/71 Group 1: *n* = 55; M/F = 18/37 Group 2: *n* = 55; M/F = 21/34	Total: N/A Group 1: 30.05 ± 6.92 Group 2: 29.74 ± 6.91	Group 1: TMD patients	Group 2: healthy subjects	Serum level of vitamin D		Analysis of the data demonstrated a significant difference in the mean serum levels of vitamin D between the study and control group (*p* = 0.001), that had an underlying prevalence of vitamin D deficiency in TMD.
Staniszewski, K. et al., 2019 [42]	Pain Research and Management	Cross-sectional study	Norway	Total: *n* = 120; M/F = 18/102 Group 1: *n* = 60; M/F = 9/51 Group 2: *n* = 60; M/F = 9/51	Total: N/A Group 1: 45 (20–69) Group 2: 46 (23–71)	Group 1: TMD patients	Group 2: healthy subjects	Serum level of hemoglobin, erythrocyte volume fraction (EVF), mean corpuscular volume (MCV), homocysteine, transferrin receptor (TfR), thyroid- stimulating hormone (TSH), free thyroxine (FT4), parathyroid hormone (PTH), cobalamin, folate, C-reactive protein, creatinine, estimated glomerular filtration rate (GFR), sodium, potassium, calcium, gamma-glutamyl transferase (GT), albumin, and 25(OH) vitamin D.		Results revealed that TMD patients had significantly higher values of hemoglobin (*p* = 0. 036), cobalamin (*p* = 0.023), albumin (*p* = 0.005), PTH (*p* = 0.038), and 25(OH) vitamin D (*p* = 0.005), and significantly lower values of creatinine (*p* = 0.006) and potassium (*p* = 0.011), compared to controls.
Yildiz, S. et al., 2020 [44]	British Journal of Oral & Maxillofacial Surgery	Prospective case-control study	Turkey	Total: *n* = 206; M/F = 74/132 Group 1: *n* = 104; M/F = 30/74 Group 2: *n* = 102; M/F = 44/58	Total: N/A Group 1: 28.64 ± 10.11 Group 2: 31.48 ± 11.33	Group 1: TMD patients (disc displacement with reduction)	Group 2: healthy subjects	VDR *BsmI* variant (after extraction of genomic DNA) and serum level of vitamin D.		Serum vitamin D level was significantly different between the patient and the control group (*p* = 0.008); particularly, vitamin D serious deficiency was more prevalent in the TMD patients (*p* = 0.00001). Logistic regression analysis revealed that the bb genotype and b allele carriers of VDR BsmI variant were significantly associated with an increased risk of disc dislocation (*p* = 0.022 and *p* = 0.01, respectively). VDR BsmI bb genotype was higher in the control group compared to the patient group (*p* = 0.045).
Yilmaz, A.D. et al., 2018 [45]	Molecular Biology Reports	Cross-sectional study	Turkey	Total: *n* = 119; M/F = N/A Group 1: *n* = 24; M/F = N/A Group 2: *n* = 25; M/F = N/A Group 3: *n* = 70; M/F = N/A	Total: N/A Group 1: 31.58 ± 8.25 Group 2: 31.8 ± 7.53 Group 2: 28.22 ± 5.9	Group 1: TMD patients (anterior disk displacement with reduction) Group 2: TMD patients (anterior disk displacement without reduction)	Group 3: healthy subjects	VDR Apa1 and Taq1 polymorphisms.		When Group 1 and Group 2 were compared to healthy subjects, Apa1 Aa genotype compared to AA genotype had odds ratios of 1.65, 1.79, and 1.64 respectively (*p* > 0.05). In TMJ-ID women versus healthy women Aa genotype had 2.06- fold (*p* = 0.15) odds compared to AA genotype. Taq1 results showed that in TMJ-ID patients and anterior disk displacement without reduction cases the Tt genotype had odds ratios of 0.63 and 0.44- fold (*p* > 0.05) respectively. In TMJ-ID women the Tt and tt genotypes had odds ratios of 0.53 and 0.73 (*p* > 0.05). Combined VDR genotypes revealed that AATT had a 3.3-fold (*p* = 1.21) odds ratio while AATt had a 2.0-fold odds ratio (*p* = 0.29) (OR 0.59, 95% CI 0.23–1.49, *p* = 0.26) compared to AaTt.

Values are presented as mean ± standard deviation and maximum–minimum (range). Abbreviations: BMD—bone mineral density; FT score—femur T score; IL-1—interleukin-1; LT score—lumbar spine T score; N/A—not applicable; NR—not reported; OA—osteoarthritis; TMD—temporomandibular disorders; TMJ—temporomandibular joint; VAS—visual analogue scale; VDR—vitamin D receptor.

**Table 3:** Joanna Briggs Institute Critical Appraisal Checklist for the studies included.

Joanna Briggs Institute Critical Appraisal Checklist for randomized controlled trials.														
Authors and Year	Q1	Q2	Q3	Q4	Q5	Q6	Q7	Q8	Q9	Q10	Q11	Q12	Q13	Total Score
Gupta et al., 2022	Y	Y	Y	Y	Y	Y	Y	Y	Y	Y	Y	Y	Y	13
Legend: Q1—Was true randomization used for assignment of participants to treatment groups?; Q2—Was allocation to treatment groups concealed?; Q3—Were treatment groups similar at the baseline?; Q4— Were participants blind to treatment assignment?; Q5—Were those delivering treatment blind to treatment assignment?; Q6—Were outcome assessors blind to treatment assignment?; Q7—Were treatment groups treated identically, other than the intervention of interest?; Q8—Was follow up complete and, if not, were differences between groups, in terms of their follow up, adequately described and analyzed?; Q9— Were participants analyzed in the groups to which they were randomized?; Q10—Were outcomes measured in the same way for treatment groups?; Q11—Were outcomes measured in a reliable way?; Q12—Was an appropriate statistical analysis used?; Q13—Was the trial design appropriate, and any deviations from the standard RCT design (individual randomization and parallel groups) accounted for in the conduct and analysis of the trial?; N—no; Y—yes; and N/A—not applicable.														
**Joanna Briggs Institute Critical Appraisal for Quasi-Experimental Studies**														
Authors and Year		Q1	Q2	Q3	Q4	Q5	Q6	Q7	Q8	Q9	Total Score			
Ahmed et al., 2017		Y	Y	N	Y	N	Y	Y	Y	Y	7			
Bashir et al., 2022		Y	Y	N	Y	N	Y	Y	Y	Y	7			
Demir et al., 2018		Y	Y	N	Y	N	Y	Y	Y	Y	7			
Hong et al., 2021		Y	Y	N	Y	N	Y	Y	Y	Y	7			
Khanna et al., 2017		Y	Y	N	Y	N	Y	Y	Y	Y	7			
Madani et al., 2019		Y	Y	N	Y	N	Y	Y	Y	Y	7			
Nemati et al., 2021		Y	Y	N	Y	N	Y	Y	Y	Y	7			
Staniszewski et al., 2019		Y	Y	N	Y	N	Y	Y	Y	Y	7			
Yildiz et al., 2020		Y	Y	N	Y	N	Y	Y	Y	Y	7			
Yilmaz et al., 2018		Y	Y	N	Y	N	Y	Y	Y	Y	7			
Joanna Briggs Institute Critical Appraisal Checklist for Case Control Studies: Q1—Is it clear in the study what is the ’cause’ and what is the ‘effect’ (i.e., there is no confusion about which variable comes first)?; Q2—Were the participants included in any comparisons similar?; Q3—Were the participants included in any comparisons receiving similar treatment/care, other than the exposure or intervention of interest?; Q4—Was there a control group?; Q5—Were there multiple measurements of the outcome, both pre and post the intervention/exposure?; Q6—Was follow up complete and, if not, were differences between groups in terms of their follow up adequately described and analyzed?; Q7—Were the outcomes of the participants included in any of the comparisons measured in the same way?; Q8—Were outcomes measured in a reliable way?; Q9—Was an appropriate statistical analysis used?; N—no, Y—yes; and N/A—not applicable.														

## Data Availability

Not applicable.
